# Characterization of the complete plastid genome of Korean endemic, *Ajuga spectabilis* Nakai (Lamiaceae)

**DOI:** 10.1080/23802359.2022.2156258

**Published:** 2023-01-15

**Authors:** Kashish Kamra, Joonhyung Jung, Hyuk-Jin Kim, Chang-Young Yoon, Joo-Hwan Kim

**Affiliations:** a Department of Life Sciences, Gachon University, Seongnam-si, Gyeonggi-do, Republic of Korea; b Korea National Arboretum, Pocheon-si, Gyeonggi-do, Republic of Korea; c Department of Biological Science, Shingyeong University, Hwaseong, Republic of Korea

**Keywords:** Plastid genome, Korean endemic, *Ajuga spectabilis*, Lamiaceae, phylogenetic analyses

## Abstract

*Ajuga spectabilis* Nakai is a Korean endemic species in Lamiaceae. In spite of its importance, genomic studies are not performed on this species. Here, we report the complete plastid genome sequences of *A. spectabilis*, which will provide valuable information for its natural conservation and future studies for the plastid genome evolution. The plastid genome is 150,417 bp in length, containing a large single-copy region (LSC) of 82,140 bp and a small single-copy (SSC) region of 17,165 bp which are separated by a pair of inverted repeats (IR) of 25,556 bp. It encodes 113 genes, including 79 protein-coding genes, 30 tRNA genes, and four rRNA genes. The overall GC content is 38.3%, and those in the LSC, SSC, and IR regions are 36.4%, 32.2%, and 43.3%, respectively, which is consistent with other *Ajuga* species. Our phylogenetic analysis revealed that *A. spectabilis* formed a close relationship with *A. ciliata* and *A. decumbens*.

## Introduction

Endemic species are crucial, as having unique genetic diversity for understanding evolution, biogeography, and speciation (Newmark and Newmark 2002; Cox et al. 2016). Various analyses have been performed to identify the plastid genome features and develop molecular markers to define the phylogenesis of endemic species (C. Kim et al. 2019; C. Kim et al. 2020). Lamiaceae Juss. are one of the largest family consisting of about 240 genera and 7000, species worldwide (Napoli et al. 2020) and is well-known for their therapeutic uses (Shinwari et al. 2013; Mamadalieva et al. 2017). Members of Lamiaceae were used in traditional medicine for circulatory, cutaneous, and musculoskeletal conditions (Zaman et al. 2022).

**Figure 1.**
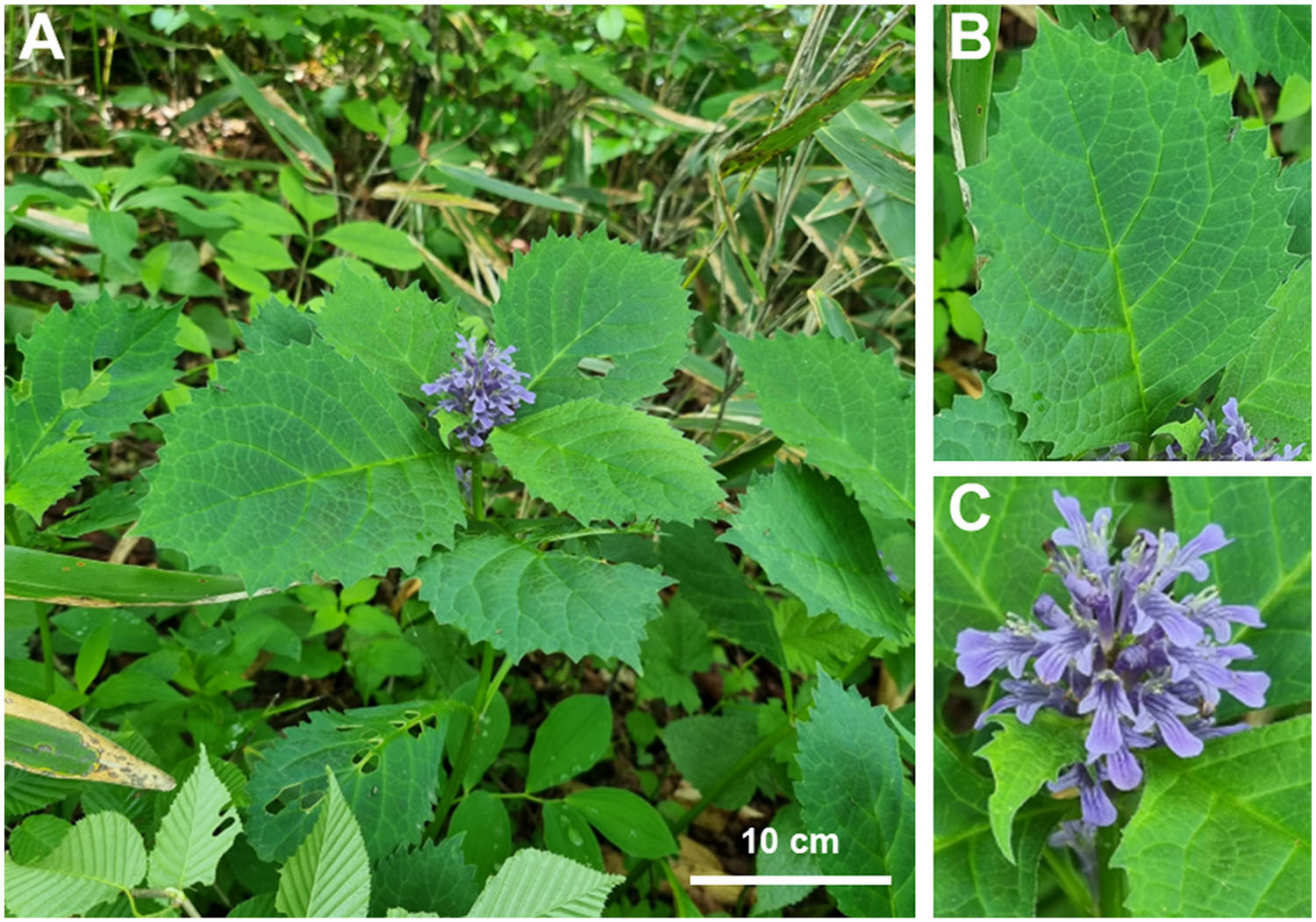
Photographs of *A. spectabilis*. (A). Perennial Plant; (B). ellipsoidal Leaves (10 cm); C. raceme inflorescence. The photographs were taken by Joonhyung Jung from Mt. Hwaya, Gapyeong-gun, Gyeonggi-do, South Korea, accessed on 20 May, 2021.

*Ajuga* is a significant genus with excellent medicinal and commercial qualities. *A. spectabilis* Nakai 1916 is an endemic perennial herb distributed in the mountains of South Korea except for Jeju Island (S.-Y. Kim et al. 2013). It is an upward-growing plant with broad ellipsoidal leaves (>8 cm in length) with deep violet flowers (Park 2007; S.-Y. Kim et al. 2013;) (Figure 1). Medicinally, it stimulates smooth and cardiac muscle (Chung et al. 1980). Although it is an endemic species with known medicinal value, its genetic data have not been examined.

Chloroplast is a plant organelle that is crucial for photosynthesis and other biochemical processes (Cheng et al. 2020; Liang et al. 2020). Chloroplast genome (plastid genome) sequences are widely analyzed due to its highly conserved structure (Ravi et al. 2008). Plastid genome with a circular structure has four regions, large single copy (LSC), small single copy (SSC), and two inverted repeats (IR). Based on the plastid genome dataset, tribal classification was proposed for 12 subfamilies of Lamiaceae (Zhao et al. 2021). Here, we assembled the plastid genome sequences of *A. spectabilis* to provide valuable information for its natural conservation and phylogenetic relationship of Korean endemic plants.

## Materials and methods

### Plant materials and DNA extraction

We collected the individual of *A. spectabilis* from Mt. Hwaya, Gapyeong-gun, Gyeonggi-do, South Korea (N 37° 42′ 04.9″, E 127° 26′ 42.9″). The voucher specimen was deposited in the Gachon University herbarium (GCU) (Joonhyung Jung, email: joonhyung0104@gmail.com) under the accession number GCU210136644. Total genomic DNA was extracted using modified 2X cetyltrimethylammonium bromide (CTAB) method (Doyle and Doyle 1987). We measured DNA concentration using spectrophotometer (Biospec-nano; Shimadzu). The DNA purity was evaluated with 1.0% agarose gel (Table S2).

### Genome sequencing, assembly and annotation

**Figure 2.**
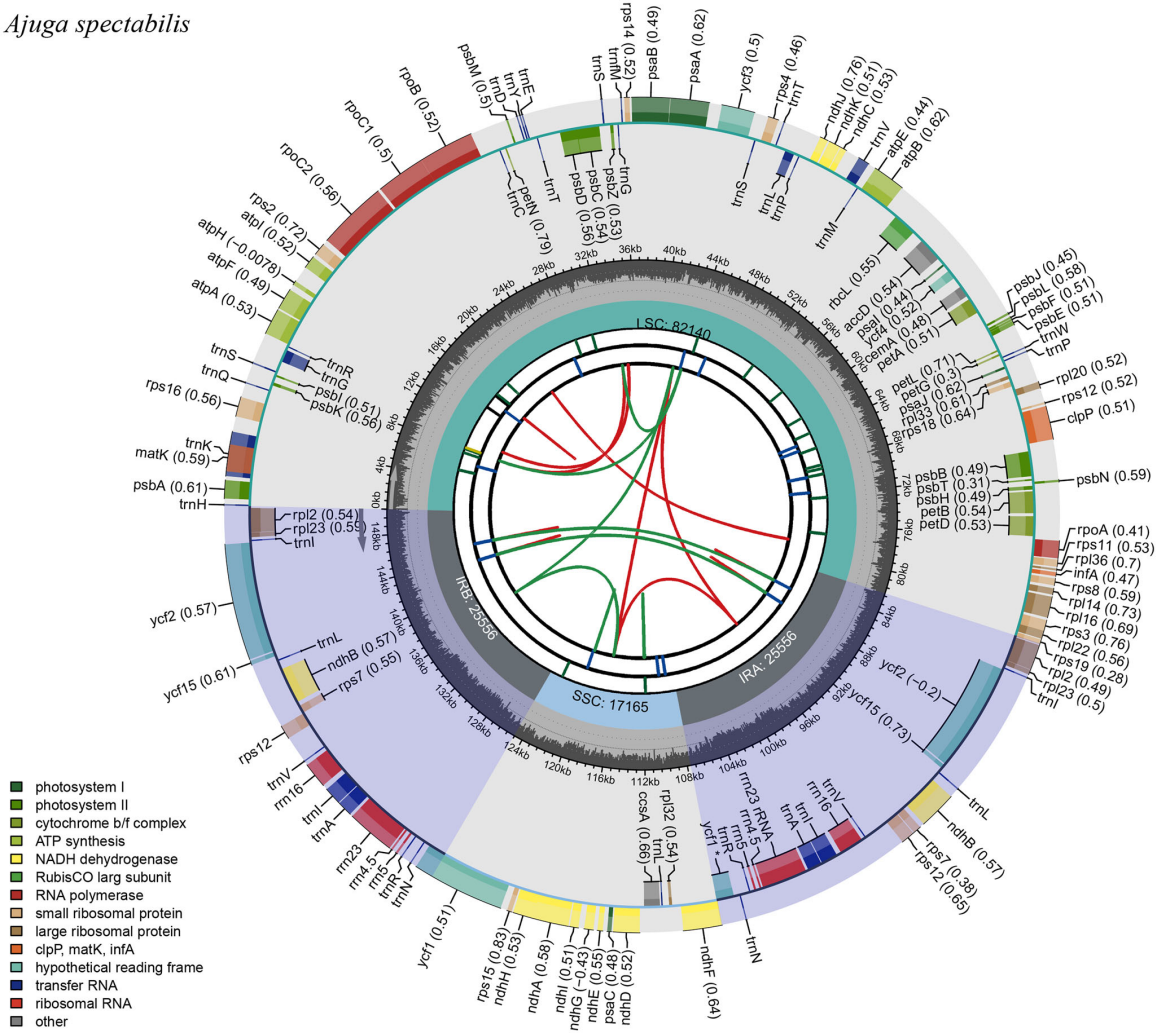
Complete plastid genome of *A. spectabilis*. The first circle shows the distributed repeats connected with red (transcribed clockwise) and green (transcribed counterclockwise) arcs from the center going outward. The second circle corresponds the tandem repeats marked with short bars. The next circle shows the microsatellite sequences as short bars. The fourth circle shows the size of the LSC and SSC. The fifth circle represents IRa and IRb. The sixth circle shows the GC contents within the plastid genome. The seventh circle defines the gene with different colors based on the functional group.

We performed the next-generation sequencing (NGS) using Illumina MiSeq Sequencer (Illumina, San Diego, California, USA). We imported the raw reads (10,781,982) and trimmed poor-quality reads using Geneious v.7.1.9 (Kearse et al. 2012). Then, we mapped to plastid genome of *A. decumbens* (GenBank accession No. MF967578). De novo assembly was implemented to generate consensus sequences and used as a reference for reassembling. We repeated this process until quadripartite structures were completed. Gaps were filled by Sanger sequencing using specific primers (Table S1). We annotated gene content and order using GeSeq (Tillich et al. 2017). All transfer RNAs were confirmed by tRNAScan-SE v.2.0 with the default search method (Lowe and Chan 2016). Complete plastid genome was illustrated using CPGview (http://www.1kmpg.cn/cpgview) (Figure 2).

### Phylogenetic analysis

We extracted a total of 79 protein-coding genes from 11 Ajugeae and two Clerodendreae species. Genes were aligned using MAFFT v.7 followed by manual modifications (Katoh et al. 2019). We performed maximum parsimony (MP), maximum likelihood (ML), and bayesian inference (BI) to reconstruct their phylogenetic relationships. Most parsimonious tree was searched with a heuristic algorithm in PAUP* v.4.0b10 (Swofford 2002). We used jModelTest v.2.1.7 (Guindon and Gascuel 2003; Darriba et al. 2012) to find the best model with Akaike’s information criterion (AIC) before running the ML and BI. The GTR + I + G was the best model for the concatenated dataset. ML analyses were implemented using the IQ-TREE web server (http://iqtree.cibiv.univie.ac.at/) (Nguyen et al. 2015). MrBayes v.3.2 was used for the BI analyses (Ronquist et al. 2012). MP bootstrap (PBP), ML bootstrap (MBP), and posterior probability (PP) were evaluated to estimate robustness for each clade. The phylogenetic trees were edited using FigTree v1.4.4 (Rambaut 2020).

## Results

The complete plastid genome of *A. spectabilis* was 150,417 bp in length, consisting of the LSC (82,140 bp) and SSC (17,165 bp) separated by a pair of IRs (25,556 bp). It encodes 130 predicted functional genes, of which 113 were unique and 17 duplicated in the IR regions. The unique genes comprised 79 protein-coding genes, 30 tRNA genes, and four rRNA genes. The overall GC content was 38.3% and in the LSC, SSC, and IRs regions were 36.4%, 32.2%, and 43.3%, respectively.

**Figure 3.**
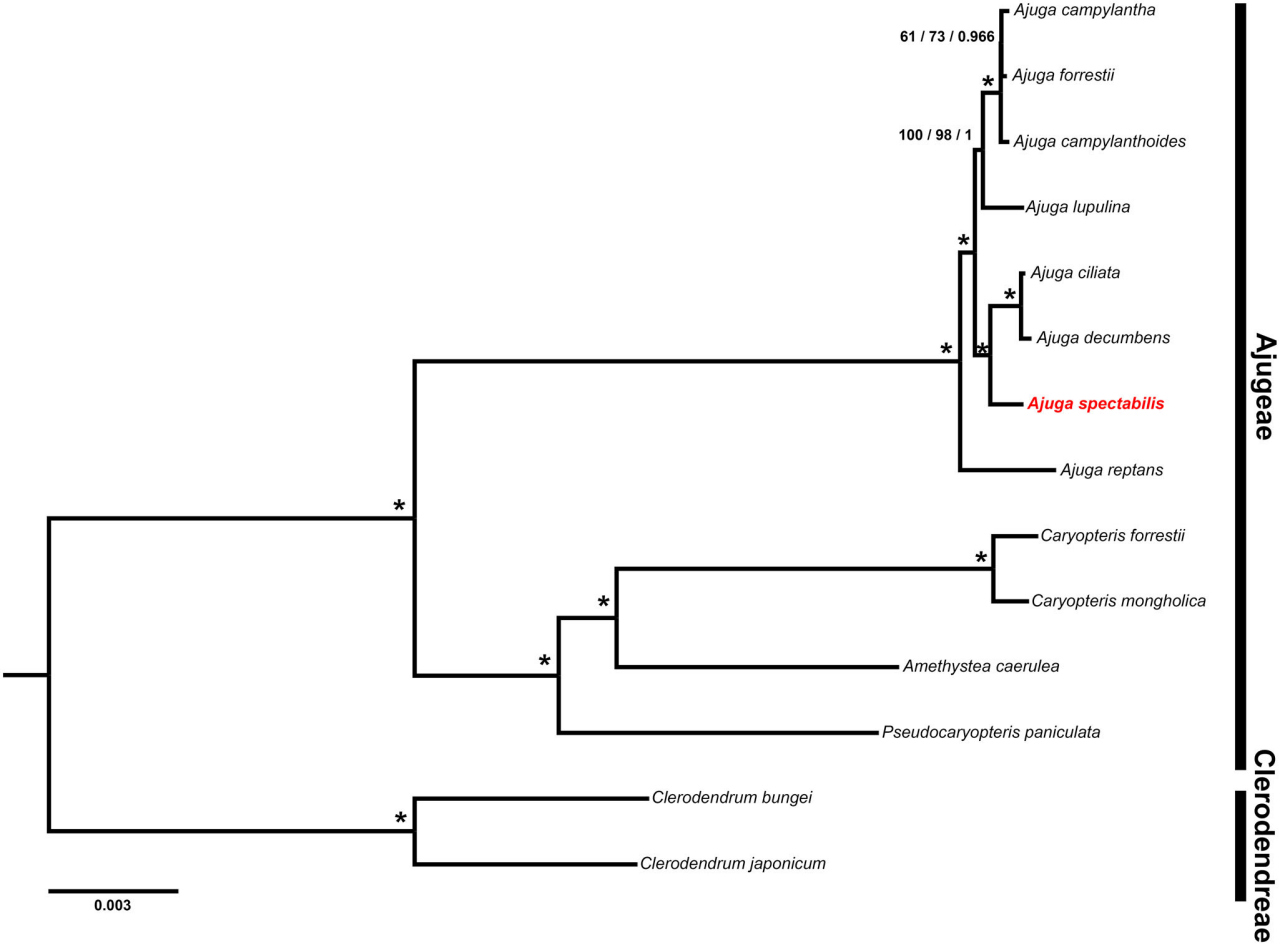
Phylogenetic tree resulting from MP, ML, and BI based on 79 plastid protein-coding genes. *A. spectabilis* is highlighted in red and the accession no. is ON620078. Numbers indicate support (PBP/MBP/PP) and asterisks near nodes indicate PBP and MBP = 100% and PP = 1.00. The GenBank accession no. of sequences used in the study are *Ajuga campylantha* MN814851; *Ajuga forresii* MN518848 (Tao et al. 2019); *Ajuga campylanthoides* MN814852; *Ajuga lupulina* MN814856; *Ajuga ciliata* MN814853; *Ajuga decumbens* MF967578; *Ajuga reptans* KF709391 (Zhu et al. 2014); *Caryopteris forrestii* MT473742 (Zhao et al. 2021); *Caryopteris mongholica* NC035729 (Liu et al. 2018); *Amethystea caerulea* MN814858; *Pseudocaryopteris paniculata* MN814866; *Clerodendrum bungei* MW242824; *Clerodendrum japonicum* MT473745 (Zhao et al. 2021).

All MP, ML, and BI trees were identical in topology (Figure 3). We identified the monophyly of Ajugeae with high support values (PBP and MBP = 100%, PP = 1.00). Also, *A. spectabilis* formed a close relationship with *A. ciliata* and *A. decumbens*.

## Discussion

Plastid genome sequences are widely used in phylogenetic implications, population genetic study, and species identification (Yang et al. 2013). Here, we assembled the complete plastid genome sequences of the Korean endemic, *A. spectabilis*. We identified that the genomic structures, gene contents and orders were highly conserved and similar to other *Ajuga* species. Our phylogenetic relationships revealed the monophyly of Ajugeae and were similar to the previous study with high support values (Zhao et al. 2021). Our results provide genetic resources for conservation and future evolutionary studies of Korean endemic species. Also, it may contribute to better resolving evolutionary relationships within phylogenetic clades of Lamiaceae.

## Supplementary Material

Supplemental Material

Supplemental Material

## Data Availability

The complete chloroplast genome sequence we obtained from this study was archived in NCBI https://www.ncbi.nlm.nih.gov/nuccore/ON620078 under accession no. ON620078. The associated Bio project, SRA, and Bio-sample numbers are PRJNA847375, SRR19592548, and SAMN28933299 respectively.
